# The Association between Sporadic Alzheimer’s Disease and the Human ABCA1 and APOE Gene Polymorphisms in Iranian Population

**Published:** 2011-04-01

**Authors:** H R Khorram Khorshid, E Gozalpour, K Kamali, M Ohadi, M Karimloo, M H Shahhosseiny

**Affiliations:** 1 Genetic Research Centre, University of Social Welfare and Rehabilitation Sciences, Tehran, Iran; 2 Reproductive Biotechnology Research Centre, Avicenna Research Institute (ACECR), Tehran, Iran; 3 Epidemiology and Biostatistics Department, University of Social Welfare and Rehabilitation Sciences, Tehran, Iran; 4 Microbiology Department, Islamic Azad University, Shahr-e-Qods Branch, Tehran, Iran

**Keywords:** Alzheimer’s disease, Genetic association, Apolipoprotein E, Polymorphism, ATP-binding cassette transporter A1, Iran

## Abstract

**Background:**

Apolipoprotein E (APOE), which its ε4 allele has been reported as a risk factor in late onset Alzheimer’s disease (AD), is the main cholesterol carrier in the brain. ATP-binding cassette transporter A1 (ABCA1) gene on chromosome 9, which has been known by genome-wide AD linkage study, has an important role in cellular cholesterol efflux. This study determines the association between sporadic AD and the human ABCA1 and APOE gene polymorphisms in Iranian population.

**Methods:**

154 AD cases and 162 control subjects from Iranian population were genotyped for APOE genotypes and ABCA1 polymorphism (R219K).

**Results:**

The frequency of ε2ε3 genotype was higher in control subjects comparing AD patients but was not significant (13% versus 5.8%) and ε3ε4 genotype frequency was significantly higher in AD cases comparing with control subjects. APOE-ε2 allele frequency in cases was lower than control subjects but this difference was not significant (4.5% versus 8%). Individuals carrying ε4 allele, developed AD 6.5 times more than non-carriers (OR=6.52, 95%CI=2.63-16.17). There was no significant association between ABCA1 polymorphism and AD.

**Conclusion:**

Unlike other studies, R219K polymorphism was not dependent on gender and APOE-ε4 allele and there was no association between APOE and ABCA1 in AD patients compared to controls.

## Introduction

Alzheimer’s disease (AD), which presents progressive cognitive defects such as memory loss, apraxia and personality changes, is the commonest cause of dementia in the mid and late ages.[1][2] Two neuropathophysiological hallmarks of AD are intracellular neurofibrillary tangles and beta amyloid plaques in brain blood vessels. As hundreds genes have been known as the risk factors for late onset AD, the well-known one is apolipoprotein E gene (APOE) which has been recognized as the most important risk factor in 65% of sporadic cases.[3] Apolipoprotein E is the main part of very low density lipoproteins, intermediate density lipoproteins (IDL), chylomicrons and the main cholesterol carrier in the brain and its synthesis is independent in central nervous system (CNS) and lung. As APOE expression is stimulated by any CNS damages or diseases, it seems that APOE regulates cholesterol metabolism and distribution in the brain to repair and stabilize neurons’ membrane and myelin.[4][5][6] Several lines of evidence show that cholesterol metabolism and Aβ deposition are related to each other. Cholesterol reducing drugs such as statin decrease brain Aβ level and increase non-amyloidogenic α -secretase cleavage of amyloid precursor protein and lead in reduced Aβ deposition.[7][8][9][10] High cholesterol diet in transgenic animal models of AD leads in increased Aβ deposition in the brain.[8][11][12] On the other hand, it was shown that the prevalence of AD is 60%-70% lower in cholesterol reducing drug consumers.[1][13][14] Thus the risk of AD and Aβ deposition can be altered by factors associated with cholesterol level.

The brain has the highest cholesterol content of the body (20%) and because of blood brain barrier, the cholesterol homeostasis is independent from cholesterol level of plasma. Most of the brain cholesterol is immobilized in the myelin and the remaining is in neurons, glials and extracellular lipoproteins.[6] Every day, 6-7 mg of excess cholesterol is converted to 24 S-hydroxycholesterol by 24 S-hydroxilase to transport out of the brain through blood brain barrier and the gene encoding 24 S-hydroxilase has found to be associated with the risk of AD.[15][16] Cellular cholesterol is transported out of the cell by a mechanism called cholesterol efflux in which a membraneassociated protein, ATP-binding cassette transporter A1 (ABCA1), transports the cholesterol to high density lipoproteins (HDL).[13] ABCA1 gene is located in 9q31.3 position which has been shown to be linked with AD by previous studies.[17][18] Loss of ABCA1 function causes Tangier’s disease which is characterized by absence of HDL, coronary artery disease and neuropathy. Like APOE, the expression of ABCA1 is regulated by RXR-LXR heterodimer and there are some evidences suggesting lack of APOE secretion from microglia, when ABCA1 is not expressed, so it seems that ABCA1 can influence APOE and cholesterol metabolism in the CNS.[5][19]

In support for a link between AD and cholesterol metabolism, it has been supposed that ABCA1 polymorphisms may influence brain cholesterol homeostasis and risk of developing AD. Association of ABCA1 polymorphisms and AD has been studied in different population and there are some positive and some negative results.[7][8][13][15][17][20][21] Raygani et al. showed that APOE- ε4 allele was a risk factor in developing AD in Iranian population but the protective role for APOE- ε2 against AD in this population was not statistically significant.[22] This study determines the association between sporadic AD and the human ABCA1 and APOE gene polymorphisms in an Iranian population.

## Materials and Methods

This case and control study involved 154 AD cases (mean age=78.55±7.80 years) and 162 control subjects (mean age=77.14±6.95 years) in which AD cases recruited using DSM IV criteria and control subjects were included if they were older than 65 years old with no known neuropsychiatry disorders. The informed consent was signed by all of them or their legal guardians. The criterion for inclusion as a case was the diagnosis of AD diagnosed by the expert psychiatrist and lacking any neurologic or psychiatric disorders for the control group. Subjects were excluded if they had any family history of dementia or neurologic diseases. AD and control subjects were recruited from Alzheimer’s society of Iran and Geriatric centers of Farzanegan, Mehrvarzan, Shayestegan, Kahrizak, Hasheminejhad and Rheumatism Center in Tehran, Iran from 2007 to 2008. The information regarding the age, sex, ethnicity, job and education were asked and recorded and finally 5 ml of peripheral blood sample was collected in tubes containing 200 μl of 0.5 M EDTA. Genomic DNA was extracted from peripheral blood leukocytes using salting-out method. APOE was genotyped by PCR-RFLP method which had been described by Wenham et al.[23] DNA was amplified by polymerase chain reaction (PCR) using forward primer: 5´-TCC AAG GAG CTG CAG GCG GCG CA-3´; and Reverse primer: 5´-ACA GAA TCC GCC CCG GCC TGG TAC ACT GCC A-3´. The 227 bp PCR products were digested by Hha I (10 U/μl, Fermentas) and loaded on a 12% polyacrylamide gel for electrophoresis; finally the gels were stained using silver staining method.

To genotype ABCA1 in AD cases and control subjects, a part of exon 7 of ABCA1 was amplified by polymerase chain reaction (PCR) using forward primer: 5´-CCT GTC ATT GTG CCT TGT G -3´; and reverse primer: 5´-GGA TTG GCT TCA GGA TGT C -3´. The 372 bp PCR product was digested by Sty I (10 U/μl, Fermentas) and loaded on an 8% polyacrylamide gel for electrophoresis; finally the gels were stained by silver staining method.

APOE and ABCA1 alleles and genotypes frequencies were analyzed through logistic regression, χ2 or Fisher’s Exact tests. Statistical significance was assumed when p value was less than 0.05. The statistical analysis and the odd ratios (OR) were determined using SPSS software (versian13, Chicago, IL, USA) and free online epidemiological software of Open Epi (2.2.1).

## Results

Distribution of age, sex, jobs, educational level and genetic background was almost the same in both groups, so there was no need to use any method for adjustment of cases and controls (Table 1). The mean age and number of females were slightly higher in patients compared to control subjects. Our data showed that the highest frequency of AD was observed in housewives and the lowest was among farmers. People with academic education had the lowest frequency among patients and illiterate individuals had the most frequency. The samples were consisted of 5 Iranian genetic backgrounds in which Fars was the most common population.

**Table 1:** Comparison of mean age, sex, jobs, education levels and genetic backgrounds between AD cases and control subjects.

**Parameter **		**AD patients (No.=154)**	**Control subjects (No.=162)**	**P value **
Age		78.55±7.80a^a^	77.14±6.95	0.091
Sex (M/F)^b^		63/91	63/99	0.714
Jobs	Housewife	55.8%	56.2%	0.938
	Own business	23.4%	21.0%	
	Worker	9.2%	8.6%	
	Farmer	3.2%	3.1%	
	Employee	8.4%	11.1%	
Education levels	Illiterate	41.6%	43.2%	0.427
	Primary school	29.2%	29.6%	
	Secondary school	16.2%	12.3%	
	Diploma	11.1%	9.3%	
	Academic	1.9%	5.6%	
Genetic background	Fars	61.0%	63.6%	0.490
	Turk	25.3%	25.3%	
	Kurd	3.9%	1.8%	
	Lor	0.7%	2.5%	
	Gilak and Mazani	9.1%	6.8%	

^a^ Mean±SD

^b^ Male/Female

The frequencies of APOE genotypes and alleles in AD cases and control subjects were shown in Table 2. The frequency of ε2ε2 genotype in control subjects was lower than that in AD cases but it was not significant (p=0.444). The distribution of ε2ε3 genotype was not significantly different in both groups (13% in controls versus 5.8% in AD, p=0.128) and OR was found to be 0.53 (95%CI=0.23-1.21). The genotype frequency of ε3ε3 was higher in control subjects compared with patients (Reference Group). The ε3ε4 genotype frequency in AD cases was significantly higher than that in control group (20.8% versus 3.7%, p=0.001).The distribution of ε2ε4 genotype was the same in both groups and different distribution of ε4ε4 genotype in the groups was not significant (2% versus 0, p=0.182).

**Table 2:** The genotype and allele frequencies were compared between AD cases and control subjects.

**Genotype/Allele**	**Alzheimer No.=154**		**Control No.=162**	**P value**	**Odds Ratio**
Genotype					
ε3ε3	69.5%		82.1%	Rf^a^	
ε2ε2	1.3%		0.6%	0.444	2.48 (0.22-27.8)
ε2ε3	5.8%		13%	0.128	0.53 (0.23-1.21)
ε2ε4	0.6%		0.6%	0.439	1.24 (0.08-20.1)
ε3ε4	20.8%		3.7%	0.001	6.52 (2.63-16.17)
ε4ε4	2%		0	0.182	undefined
Allele					
ε3	82.8%		90.1%	Rf^a^	
ε2	4.5%		8%	0.243	0.67 (0.34-1.32)
ε4	12.7%		1.9%	0.001	7.44 (3.1-17.9)

^a^ Reference Group.

The APOE-ε4 allele frequency was significantly higher in AD cases compared with control subjects (12.7% versus 1.9%, p=0.001). Comparing allele frequency in APOE- ε4 allele carriers with non-carriers, OR was found to be 6.52 (95%CI=2.63-16.17). The frequency of APOE-ε3 allele in patients was lower than that in control group (Reference Group). Despite of higher APOE-ε2 allele frequency in AD cases compared with control subjects, this difference was not statistically significant (p=0.243 and OR=0.67, 95%CI=0.34-1.32) (Table 2).

Table 3 shows APOE genotype and allele frequencies distributed by sex groups. ε2ε3 genotype frequency in control subjects was higher than AD subjects in men and women group (p>0.05). The genotype frequency of ε3ε4 in AD cases was higher than control subjects in both male and female groups but it was significant just in female group (p=0.001). The frequency of APOE-ε4 allele in patients was significantly higher than control subjects in both males and females with different OR [males: p=0.002, OR=8.3 (1.86-37); females: p=0.001, OR=5.59 (2.07-15.05)].

**Table 3:** APOE genotype and allele frequencies distributed by sex.

**Genotype/Allele**	**Female**	**Male**
ApoE genotypes		
ε3/ε3	Rf^a^	Rf^a^
ε2/ε3	P=0.522, OR=0.663 (0.22-1.75)	P=0.104, OR=0.23 (0.05-1.13)
ε3/ε4	P=0.001, OR=7.86 (2.58-23.9)	P=0.080, OR=4.7 (0.96-22.8)
ε4/ε4	No data	P=0.319, OR= undefined
APOE alleles		
ε3	Rf^a^	Rf^a^
ε4	P=0.001, OR=5.59 (2.07-15.05)	P=0.002, OR=8.3 (1.86-37)
ε2	P=0.157, OR=0.46 (0.17-1.19)	P=0.878, OR=0.8 (0.29-2.24)

^a^ Reference Group

The genotypes and alleles of ABCA1 gene was compared in two groups of AD cases and controls. As it has been summarized in Table 4, the GG genotype frequency was not different between AD cases and controls (Reference Group). Comparing GA and AA genotype frequencies, there was no significant difference between AD cases and control subjects (GA: p=0.451 and AA: p=0.696). No significant difference was observed between allele frequencies in cases and controls (p=0.592). Examining data stratified by gender, neither female AD cases nor male AD cases showed significant genotype and allele frequencies compared with female and male controls.

**Table 4:** Genotypes and allele frequencies in AD cases and control subjects by sex.

	**Total**			**Female**			**Male**		
**Genotype**	**AD (No.=154)**	**Control (No.=162)**	**P value**	**AD (No.=91)**	**Control (No.=99)**	**P value**	**AD (No.=63)**	**Control (No.=63)**	**P value**
GG	33.1%	37%	Rf^a^	9.6%	39.4%	Rf	23.8%	33.3%	Rf
GA	48.7%	45.1%	0.451	0.4%	40.4%	0.803	55.6%	52.4%	0.341
AA	18.2%	17.9%	0.696	16.4%	20.2%	0.487	0.6%	14.3%	0.197
Allele									
G	57.5%	59.6%	0.592	59.6%	61.5%	0.699	51.6%	59.5%	0.205
A	42.5%	40.4%		40.4%	38.5%		48.4%	40.5%	

^a^ Reference Group

Furthermore, stratification of data by ε4 allele of APOE, which had been genotyped for the AD cases and control subjects in the previous study, did not change the results. ABCA1 genotypes and alleles of AD and control subjects were compared between ε4 carriers and ε4 non-carriers (Table 5). Distribution of GG, GA and AA genotypes were not significantly different between AD cases and control subjects of ε4 carriers and non-ε4 carriers.

**Table 5:** ABCA1 genotypes and alleles distribution in APOE ε4 carriers and APOE ε4 non-carriers.

	**APOE ε4 carriers**			**APOE ε4 non-carriers**		
**Genotype Frequency**	**Controls (No.=7)**	**Patients (No.=36)**	**P value**	**Controls (No.=155)**	**Patients (No.=118)**	**P value**
GG	42.9%	25%	Rf^a^	36.8%	35.6%	Rf
GA	42.9%	58.3%	0.618^b^	45.2%	45.8%	0.866
AA	14.2%	16.7%	1^b^	18%	18.6%	0.854
Allele Frequency						
G	64.3%	54.2%	0.485	64.3%	54.2%	0.836
A	35.7%	45.8%		35.7%	45.8%	

^a^ Reference Group

^b^ Fischer exact test p value

## Discussion

According to this study, APOE-ε4 allele is a risk factor for developing late onset AD in Iranian population like many other populations.[24][25][26][27][28][29] Although ε2ε3 genotype seems to play a protective role against AD but the protective role of APOE- ε2 allele has not demonstrated in this study and it may be proved by a larger sample size.

The risk of developing AD in individuals with ε2ε3 genotype is about 0.53 (95%CI=0.23-1.21) compared with individuals without this genotype so ε2ε3 genotype seems to be protective against AD whereas protective role of ε2 allele has not demonstrated in Iranian population yet. APOE-ε4 allele carriers develops AD, 6.5 times more than non-carriers (6.52, 95%CI=2.63-16.17). This allele’s risk seems different in males and females. Different OR for ε4 allele in men and women indicates that risk of AD in male APOE-ε4 allele carriers (OR=8.421, CI=1.894-37.44) is higher than female carriers (OR=5.846, CI=2.173-15.73), so it seems that despite the agedependant and dosage-dependent manner of this allele which were investigated in Iranian population by Raygani et al.,[22] it may act in a sex-dependent way as well. As three patients were observed with ε4ε4 genotype, it was not possible to assess the dosagedependent action of ε4 allele in this study.

As the study groups were similar based on potential confounders (age, sex, genetic background, job and education), it can be assumed that the results are mainly unbiased. There was no reliable history or evidence for the time of AD onset, so we couldn't evaluate the effect of different genotypes or alleles on the age of onset in the AD subjects. In an autopsybased study, the frequency of ε4 allele and ε4ε4, ε3ε4 genotypes were 40%, 16.5% and 43.2% in AD patients and 16%, 2.2 and 20.9% in the control group.[30] In a group of African Americans AD patients, a significantly increased risk of AD was associated with two ε4 alleles or one ε4 allele when compared to ε3ε3 genotype.[31] In our study, the frequencies of ε4 allele and ε4ε4, ε3ε4 genotypes were lower than results of Raygani et al.,[22] but the proportion of them was the same and the results of two studies in Iranian population are consistent. No significant association was found between ε2 allele or related genotypes and AD but it sounds to work as protective factor for AD; however this finding, should be confirmed in further studies with a larger sample size .

In initial studies, it had been shown that ABCA1 expression can affect the level of APOE expression. Increased amyloid deposition in ABCA1 null mice and position of this gene on chromosome 9, which was linked to AD, were another evidence to confirm ABCA1 gene as a target gene for AD association studies.[3][5] In 2003, Wollmer reported that A allele of ABCA1 causes 1.7 years delay at onset of AD and A allele carriers had 33% lower cholesterol in cerebrospinal fluid, than non-carriers of this allele.[15] Significant increased frequency of A allele in control subjects compared with AD cases in Chinese population was reported by Wang et al. in 2007, while Sunder et al. reported that AD incidence is 1.5 times more frequent in A allele female carriers compared with female non-carriers in white American population. [8][13]

In this study, ABCA1 and its association with AD was studied in Iranian population for the first time. No association was observed between genotypes and alleles of ABCA1and AD. In 2006, Shibata et al. reported that ABCA1-A allele frequency was significantly higher in APOE-ε4 carriers compared with ε4 non-carriers.[7] Therefore it was decided to investigate about this finding, but stratification by gender and APOE-ε4 allele did not change the result and there was no association between genotypes and alleles of ABCA1 and the risk of AD among APOE-ε4 carriers and non-carriers.
